# Collagen Type I alpha1 (COL1A1) Gene Polymorphism and Bone Mineral Density in Postmenopausal Kazakh Women

**DOI:** 10.5195/cajgh.2014.144

**Published:** 2014-12-12

**Authors:** Akbota Aitkulova, Ainur Akilzhanova, Zhannur Abilova, Zaida Zhumatova, Gulbanu Akilzhanova, Elena Zholdybayeva

**Affiliations:** 1 National Center for Biotechnology, Astana, Kazakhstan; 2 Center for Life Sciences, Nazarbayev University, Astana, Kazakhstan; 3 City Hospital #1, Pavlodar, Kazakhstan; 4 Center for Perinatology, Pavlodar, Kazakhstan

**Keywords:** osteoporosis, bone mineral density, gene polymorphism

## Abstract

**Introduction:**

Single nucleotide polymorphism (SNP) at the collagen type I alpha 1 gene (COL1A1) rs1800012 has been widely studied and has shown an association with bone mineral density (BMD) and fractures. A minor allele TT of this SNP was found to be greatly overrepresented in individuals with fractures compared to controls, thus becoming a good predictor of increased fracture risk. The aim of this investigation was to evaluate potential association between COL1A1 gene polymorphism and osteoporosis in Kazakh postmenopausal women.

**Methods:**

The study population included 103 postmenopausal women recruited from Pavlodar and Almaty clinics. BMD was measured using DEXA. Genomic DNA was extracted from peripheral venous blood of study participants with Wizard® Genomic *DNA Purification Kit* (Promega, USA). Detection of COL1A1 +1245G/T (Sp1) polymorphism was done by the TaqMan® SNP Genotyping Assay of real-time PCR.

**Results:**

Densitometry results revealed 36 osteoporotic, 42 osteopenic, and 25 normal postmenopausal women. Data analysis of 1245G>T polymorphism in COL1A1 gene in the group of women with osteopenia and osteoporosis revealed deviation from Hardy-Weinberg equilibrium. The mutant TT genotype was prevalent compared to the heterozygous genotype GT in both groups. Distributions were 83% GG, 3% GT, and 14% TT in the group with osteopenia and 80% GG, 6% GT, and 14% TT in the group with osteoporosis. The distribution of genotypes frequency in the group of normal postmenopausal women was 76% GG, 16% GT, and 8% TT.

**Conclusion:**

These results suggest that TT genotype of COL1A1 +1245G/T (Sp1) polymorphism is associated with risk of postmenopausal osteoporosis in Kazakh women. Further studies involving a larger number of women are needed to clarify the relationship of this polymorphism with risk of osteoporosis.

